# High-content tripartite split-GFP cell-based assays to screen for modulators of small GTPase activation

**DOI:** 10.1242/jcs.210419

**Published:** 2018-01-01

**Authors:** Faten Koraïchi, Rémi Gence, Catherine Bouchenot, Sarah Grosjean, Isabelle Lajoie-Mazenc, Gilles Favre, Stéphanie Cabantous

**Affiliations:** 1 Cancer Research Center of Toulouse, INSERM U1037, 31037 Toulouse, France; 2 Université de Toulouse, Toulouse, France

**Keywords:** GTPase activation, Split-GFP, Protein–protein interaction, GEF, Guanine nucleotide exchange factor, Cell-based assays, High-content analysis

## Abstract

The human Ras superfamily of small GTPases controls essential cellular processes such as gene expression and cell proliferation. As their deregulation is widely associated with human cancer, small GTPases and their regulatory proteins have become increasingly attractive for the development of novel therapeutics. Classical methods to monitor GTPase activation include pulldown assays that limit the analysis of GTP-bound form of proteins from cell lysates. Alternatively, live-cell FRET biosensors may be used to study GTPase activation dynamics in response to stimuli, but these sensors often require further optimization for high-throughput applications. Here, we describe a cell-based approach that is suitable to monitor the modulation of small GTPase activity in a high-content analysis. The assay relies on a genetically encoded tripartite split-GFP (triSFP) system that we integrated in an optimized cellular model to monitor modulation of RhoA and RhoB GTPases. Our results indicate the robust response of the reporter, allowing the interrogation of inhibition and stimulation of Rho activity, and highlight potential applications of this method to discover novel modulators and regulators of small GTPases and related protein-binding domains.

## Abstract (precis)

**Summary:** The development of a fluorescent reporter of GTPase activation based on tripartite split-GFP that enables the evaluation of GEF activity and the effect of modulators of GTPase activation in a high-content analysis.

## INTRODUCTION

Small GTPases of the Ras superfamily are crucial players of cell signal transduction that link extracellular receptor sensing to downstream signaling responses. Among the Ras superfamily, Rho members are key regulators of fundamental cellular processes such as cell adhesion and motility, gene expression and cell proliferation (Jaffe and Hall, 2005). Consequently, deregulation of Rho GTPases expression and activity levels contributes to tumorigenesis, including oncogenic transformation, cell survival and metastasis (Porter et al., 2016; Vega and Ridley, 2008). Small GTPases function like molecular switches that alternate between a GTP-bound active state and a GDP-bound inactive conformation. Binding to GTP is promoted by Rho guanine nucleotide exchange factors (GEFs) that promote binding of intracellular GTP onto the GTPase. Hydrolysis is catalyzed by GTPase-activating proteins (GAPs), which terminates their activity cycle. It is only in their active state that GTPases interact with a range of different effectors to drive their function (Ellenbroek and Collard, 2007; Vega and Ridley, 2008).

RhoB belongs to the RhoA family of GTP-binding proteins, which regulates gene expression, actin-based cell motility (Heasman and Ridley, 2008) and, importantly, the balance between cellular survival and cell death (Fritz and Kaina, 2000; Meyer et al., 2014; Milia et al., 2005). Unlike its homologs RhoA and RhoC, RhoB acts as a tumor suppressor (Liu et al., 2001) and its expression is downregulated in various cancers (Adnane et al., 2002; Calvayrac et al., 2014; Mazieres et al., 2004). Recent studies have highlighted a novel role of RhoB in critical biological processes such as angiogenesis (Gerald et al., 2013), the response to DNA damage (Mamouni et al., 2014; Wang et al., 2014) and the regulation of epithelial-mesenchymal transition (Bousquet et al., 2015). Furthermore, we have shown that RhoB modulates the response to anticancer therapeutics, thereby promoting resistance following the inhibition of receptor tyrosine kinase signaling (Calvayrac et al., 2017; Delmas et al., 2015). Therefore, understanding mechanisms of RhoB activation and modulating its function would provide novel strategies to current therapeutics.

To monitor small GTPase activation, a general strategy consists in evaluating the binding of the GTP-bound active form in the presence of a GTPase-binding domain of specific effectors proteins immobilized on beads (Benard and Bokoch, 2002; Chuang et al., 1994; Ren et al., 1999). Despite its convenient use at the bench, this method is limited by the labile nature of GTP-bound proteins in cell extracts, which requires cautious processing steps and is therefore limited by a low throughput. Moreover, small GTPase activation is tightly regulated by the subcellular localization of GTPase regulators and effectors (Ellenbroek and Collard, 2007), which requires the development of new approaches that report these mechanisms in live cells. Live-cell assays using fluorescence resonance energy transfer (FRET) sensors have greatly contributed to highlight the spatiotemporal activity of several Rho GTPases from the RhoA subfamily, including RhoB (Pertz et al., 2006; Reinhard et al., 2016). In these sensor systems, two cyan and yellow fluorescent protein derivatives are covalently bound to the Rho GTPase and its effector, such that Rho GTPase activation will promote Rho-binding domain (RBD)–Rho-GTP binding leading to an energy transfer from CFP to YFP. Such approaches highlighted the fine-tuning kinetic and spatial control of GTPase activation upon stimuli, revealing both GTPase activation and inactivation processes. Nevertheless, FRET assays require careful optimization of sensor FRET pairs and significant image processing work that can limit their use for high-content assays (Pietraszewska-Bogiel and Gadella, 2011). Here, we adapted the tripartite split-GFP system (triSFP) to develop a sensor of GTPase–effector interaction that is suitable for the throughput of high-content screening to evaluate modulators of small GTPase activation. The system uses small GFP tags (strands β10 and β11) as fusion partners of the GTPase and their binding domain that provide a minimum interference on passenger proteins and a great modularity in biosensor design. Detection of GTPase association is monitored by a third split-GFP detector fragment (GFP strands β1–β9), which confers a strong specificity with low background signals in living cells (Cabantous et al., 2013).

By using various members of Rho and Ras family GTPases as models, we validated the triSFP system as a fluorescent reporter of the GTP-bound active state of small GTPases and developed a quantitative analysis of GTPase–effector interactions in live cells. To monitor GTPase activation with a higher throughput, we engineered an optimized cellular model that shows a robust response to the modulation of RhoB activation upon GEF downregulation and Rho inhibitor treatment. In a high-content imaging analysis, we evaluated the efficiency of several cytoskeletal poisons on RhoA and RhoB GTPases. Our results indicate a good correlation between Rho activation and microtubule depolymerization, thus demonstrating the sensitivity of the assay to report intracellular mechanisms that regulate Rho activity.

## RESULTS

### Characterization of triSFP to monitor the GTPase active form

**Fig. 1.**
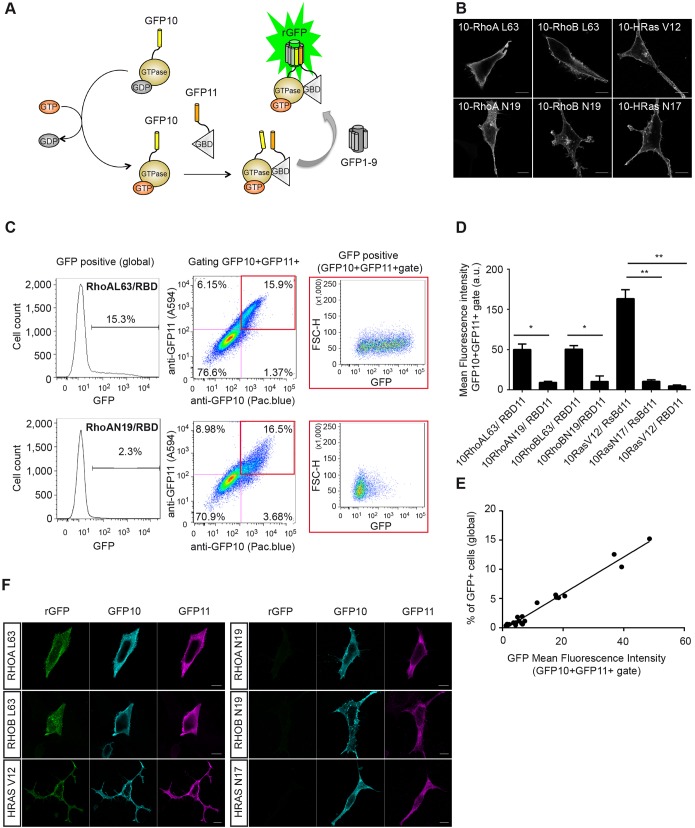
**Characterization of a split-GFP reporter of small-GTPase activation.** (A) Design of the reporter system. The GTPase is fused to the C-terminus of GFP10 and the cognate binding domain of the GTPase effector (GBD) to the N-terminus of GFP11. When the GTPase is activated (GTP-bound), interaction with the effector domain occurs, enabling complementation with GFP1-9 and formation of reconstituted full-length GFP (rGFP). (B) Localization of the indicated GFP10–Rho and GFP10–Ras mutants in HEK_GFP1-9 cells (anti-GFP10 immunostaining). L63 and V12 mutants are constitutively active; N19 and N17 mutants are dominant negative. Scale bars: 10 µm. (C) FACS analysis of active GFP10–RhoA (L63) and inactive GFP10–RhoA (N17) mutants with the GFP11-tagged Rho-binding domain of Rhotekin (RBD-11). Representative dot plots show the gating strategy to quantify GFP fluorescence from the global population and from the cell population positive for GFP10 and GFP11 immunostaining (GFP10+ GFP11+). The histograms on the left show the percentage of GFP-positive cells relative to untransfected cells in the global population. See also Fig. S2. (D) Quantification of split-GFP fluorescence in the GFP10+GFP11+ region from HEK_GFP1-9 cells co-transfected with the indicated GFP10–Rho and GFP10–HRas variants and the respective GTPase binding domain of their effector. RsBD-11, H-Ras-binding domain of c-Raf1. Results are mean±s.e.m.; *n*=3 independent experiments. *P<0.05, **P<0.01 (paired Student's *t*-test). (E) Correlation between the percentage of GFP fluorescent cells in the global population and the mean GFP fluorescence intensity of GFP10 and GFP11 co-expressing cells for the set of GTPase–effector interactions tested above. (F) Representative confocal microscopy images of Rho–RBD and Ras–RsBD interactions leading to fluorescence of rGFP, as well as immunofluorescence from anti-GFP10 (cyan, GTPase) and anti-GFP11 (magenta, GBD) antibody staining. Scale bar: 10 µm.

To monitor the GTPase active form with triSFP, we designed a model in which the GTPase is C-terminally fused to GFP β-strand 10 M2 (GFP10), and the cognate GTPase-binding domain (GBD) of the GTPase effector is N-terminally fused with GFP β-strand 11 M4 (GFP11) (Fig. 1A; Fig. S1A). When the GTPase is activated (GTP-bound), interaction with the effector domain occurs, enabling complementation of the tethered GFP10 and GFP11 tags with GFP1–9 and formation of reconstituted full-length GFP (rGFP) (Fig. 1A).

We first exemplified our experimental approach by using two human Rho homologs, RhoA and RhoB, that present 85% sequence identity but display different subcellular localizations and regulate distinct signaling pathways (Wheeler and Ridley, 2004). We first verified that the GFP10 tag did not perturb the interactions of the wild-type and mutated forms of Rho proteins to their effector domain *in vitro.* Indeed, we observed appropriate binding of GFP10–Rho chimera from cell extracts to GST–RBD beads according to their activity state (Fig. S1B). We extended our validation to other members of the Ras superfamily by fusing constitutively activated (V12) and dominant-negative (N17) mutants of HRas to GFP10, and generating C-terminal GFP11 fusions with the Ras-binding domain (RsBD) of the effector Raf-1 (Chuang et al., 1994) or with the RBD of rhotekin (Ren et al., 1999) (see Materials and Methods and Fig. S1A). Because no commercial antibody was available to detect strands β10 and β11 of these engineered variants, we developed polyclonal antibodies that specifically distinguish GFP10 (rabbit serum) and GFP11 (rabbit and mouse sera) fragments (Fig. S1C). Immunofluorescence of HEK cells transfected with GFP10–Rho and GFP10–HRas fusions indicated localization patterns of GTPase protein fusions that correlated with their expected subcellular localizations, mostly at the plasma membrane for constitutively activated mutants, and a more significant intracellular staining for GDP-bound forms (Michaelson et al., 2001) (Fig. 1B), confirming the absence of interference from the GFP10 tag on the intracellular targeting of small GTPases.

We then evaluated how the split-GFP reporter fluorescence correlates with the activity of various Rho and Ras mutants. To accurately quantify GTPase–effector interactions by flow cytometry after transient transfection, we investigated an approach that combines the detection of both split-GFP complementation fluorescence and expression levels of GFP10 and GFP11 fusion proteins (Fig. 1C). Plasmid vectors encoding for GFP10–Rho and GFP10–HRas fusions with their cognate effector domains RBD–GFP11 and RsBD–GFP11 were transfected in HEK_GFP1-9 cells that stably express the GFP1–9 fragment (Cabantous et al., 2013). At 16 h after transfection, fixed cells were stained with rabbit anti-GFP10 and mouse anti-GFP11 antibodies followed by secondary labeling with compatible dyes (Pacific Blue for GFP10, Alexa Fluor 594 for GFP11) (Fig. 1C; Fig. S2A,B). A total of 5000 to 10,000 cells were collected in the gating region corresponding to GFP10- and GFP11-positive staining, which was further used to calculate the GFP mean fluorescence intensity (Fig. 1C,D). Quantification of triSFP reporter intensities in GFP10+ and GFP11+ gating regions indicated a 5-fold increase in mean fluorescence intensities of cells co-expressing constitutively active GFP10–RhoAL63 and RBD–GFP11, and GFP10–RhoBL63 and RBD–GFP11 compared to cells that express their dominant-negative counterparts, while HRas mutants exhibited a 12-fold change between their active and inactive forms (Fig. 1D). Considering that acquisition was performed in a gating region that corresponded to the same expression levels of Rho and Ras mutants, it is likely that such differences can be attributed to variability in GTPase–effector affinities in live cells (Fig. S2A). Indeed, for constitutively activated GTPase variants, the percentage of GFP-positive cells in the GFP10+ and GFP11+ region was in the same range as for the GFP10–zipper–GFP11 domain that spontaneously associates with GFP 1–9 (Fig. S2C). Dominant-negative GTPase variants exhibited mean fluorescent intensities for the GFP10+ and GFP11+ cells that were close to background levels (Fig. 1C,E; Fig. S2A), indicating that split-GFP complementation is negligible for the inactive form. Furthermore, co-expression of the active GFP10-HRas V12 mutant with the unrelated Rhotekin-RBD–GFP11 did not produce GFP fluorescence, which confirms the robustness of the assay for detecting specific GTPase–effector interactions (Fig. 1D). Missing one of the split-GFP tagged domains abolished GFP reconstitution, and specific recognition of the corresponding fusion proteins was observed when anti-tag antibodies were combined in double immunostaining conditions (Fig. S2D). From the three independent experiments, we observed a linear correlation between the percentage of GFP fluorescent cells in the global population and the GFP fluorescence of GFP10 and GFP11 co-expressing cells, indicating that either parameter may be used as indicator of positive interaction in the split-GFP assay (Fig. 1E). We next verified that discrimination between the active and inactive GTPase could be robustly visualized by fluorescence microscopy. The same constructs as above were transiently expressed in HEK_GFP1-9 cells that were immunostained with anti-GFP10 and anti-GFP11 antibodies with compatible dyes to correlate the subcellular localization and expression of GFP10- and GFP11-tagged protein domains with that of the triSFP activity reporter (Fig. 1F). Supporting the flow cytometry analysis (see Fig. 1D), split-GFP complementation (rGFP) correlated with the coexpression of active GTPase mutants while no GFP fluorescence was detected with dominant-negative variants (Fig. 1F). Taken together, these results indicate that the fluorescence in the triSFP Rho activation assay is correlated with the level of the GTP-bound active forms of small GTPases.

### Improving split-GFP fluorescence with anti-GFP nanobody

**Fig. 2.**
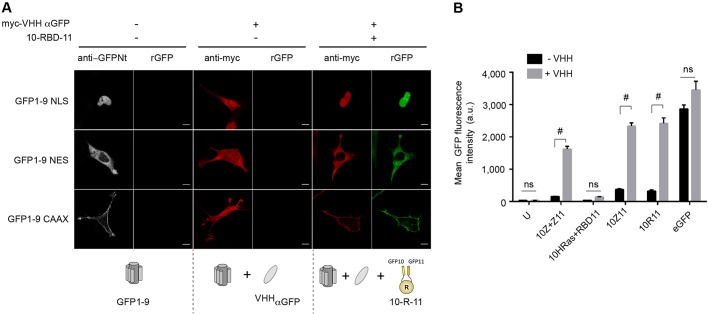
**An anti-GFP nanobody binds to the rGFP and boosts split-GFP fluorescence.** (A) Left panels, representative images of GFP1-9 localization (anti-GFPNt antibody staining, gray) for GFP1-9 variants targeted to the nucleus (NLS), cytoplasm (NES) and the plasma membrane (CAAX). Localization of the anti-GFP VHH nanobody (anti-Myc staining, red) with GFP1-9 variants alone (middle panels), and in the presence of a self-assembling GFP10–RBD–GFP11 (10-R-11) domain that induces split-GFP complementation (right panels). Scale bars: 10 μm. (B) Quantification of split-GFP fluorescence intensity of MRC5-SV_GFP1-9 cells expressing (+) or not (−) the VHH domain and transfected with the indicated constructs: interacting leucine zippers (10Z+Z11), non-interacting 10HRas and RBD11, and GFP1-9 self-associating domains [GFP10–Zipper–GFP11 (10-Z-11) and GFP10–RBD–GFP11 (10-R-11)]. U, untransfected cells. Mean fluorescence intensity was quantified in GFP10+GFP11+ expressing cells after double immunostaining with anti-GFP10 and anti-GFP11 antibodies; full-length eGFP fluorescence was collected in the whole cell population. Results are mean±s.e.m.; *n*=3 independent experiments. ^#^*P*<0.0001; ns, not significant (multiple *t*-tests using the Holm–Sidak method with α=5%).

Overexpression of GTPases induces strong cell toxicity and may alter the balance with regulators that lead to a constitutively activated phenotype. Knowing that lowering expression of GFP10–GTPase and GBD–GFP11 fusions leads to a decrease of split-GFP fluorescence, we evaluated how a GFP intrabody would improve the resulting fluorescence signal. One single-domain antibody based on camelid heavy-chain antibodies (VHH or nanobody) was engineered to boost GFP fluorescence by modulating the spectral properties of wild-type GFP (Kirchhofer et al., 2010). To investigate whether the anti-GFP VHH binds solely to the reconstituted tripartite split-GFP and not to the GFP1-9 scaffold, we analyzed the localization of the nanobody with GFP1-9 or with reconstituted GFP. To achieve that, we designed several GFP1-9 variants tagged with subcellular localization signal sequences: a nuclear localization sequence (NLS) for nuclear delivery; a nuclear export sequence (NES) for the cytoplasm, and fused to a CAAX sequence for plasma membrane targeting (Fig. 2A). Co-expression of these GFP1-9 variants with the anti-GFP VHH did not affect the cytoplasmic subcellular localization of the anti-GFP intrabody in the cell (Fig. 2A), indicating that no specific binding of the anti-GFP intrabody to GFP1-9 variants occurred. In contrast, concomitant expression of the non-prelocalized RBD domain fused to GFP10 and GFP11 (10-R-11) induced split-GFP complementation at the GFP1-9-targeted compartment and resulted in the colocalization of the anti-GFP VHH with reconstituted GFP (Fig. 2A). These data clearly indicate that the anti-GFP VHH solely binds to the reconstituted split-GFP and not to GFP1-9 alone.

To evaluate the enhancement of the anti-GFP VHH on split-GFP fluorescence, we performed FACS quantifications on previously described interacting domains, such as the GCN4 leucine zipper [denoted with a Z; i.e. interaction between GFP10-Z and Z-GFP11 (10Z+Z11)] (Cabantous et al., 2013), the non-interacting GFP10–HRas V12 and RBD–GFP11, as well as GFP1-9 self-assembling domains for the RBD (10-R-11) and the GCN4 zipper (10-Z-11). Those constructs were expressed in immortalized human pulmonary fibroblasts that stably express GFP1-9 alone (MRC5_GFP1-9) or in the presence of the anti-GFP VHH (MRC5_GFP1-9_VHH). Flow cytometry analysis of transiently transfected plasmid fusions indicated a 5–8-fold increase in the split-GFP fluorescence intensity of cells expressing the anti-GFP VHH (+VHH) compared to those that do not express the intrabody (–VHH) (Fig. 2B). Double immunostaining with anti-GFP10 and anti-GFP11 antibodies validated close expression levels of GFP10- and GFP11-tagged domains in both cell lines (Fig. S3). The fluorescence increase was observed both with GFP10- and GFP11-positive interactions (10Z+Z11) and with bimolecular complementation of GFP1-9 with self-assembling domains. More importantly, it did not affect significantly fluorescence levels from untransfected cells or from cells transfected with the non-interacting GFP10-HRas and RBD-11 pair, indicating that the anti-GFP VHH enhancer did not induce artifactual fluorescence or forced split-GFP association.

### Developing a cell-based model to monitor RhoB activation

**Fig. 3.**
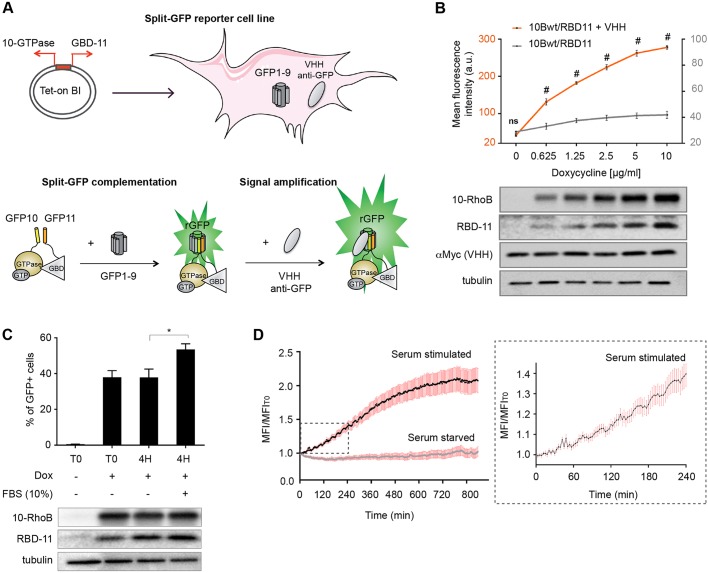
**triSFP RhoB activation reporter cell model.** (A) A bidirectional inducible promoter vector co-expressing GFP10–Rho and RBD–GFP11 chimera was used to transduce MRC5-SV fibroblasts expressing the GFP1-9 fragment and the VHH anti-GFP nanobody. Binding of the nanobody to rGFP enhances its fluorescence. (B) Doxycycline dose–response rGFP fluorescence in MRC5_GFP1-9 in the presence (+VHH) or in the absence (−VHH) of the anti-GFP intrabody. Results are mean±s.e.m.; *n*=3 independent experiments. #*P*<0.0001; ns, not significant (Holm–Sidak *t*-test with α=5%). Control of protein expression is shown in the western blot below the graph (anti-GFP10 and anti-GFP11 antibodies; anti-Myc for VHH). (C) Flow cytometry analysis of serum-induced activation. RhoB activation reporter was expressed with 2.5 µg/ml doxycycline (Dox+), and the cell line was subjected or not to serum stimulation (4 h) after a starvation period of 48 h (T0). Results are mean±s.e.m.; *n*=3 independent experiments. *P<0.05 (paired Student's *t*-test). The immunoblot below the graph shows GFP10–RhoB and RBD–GFP11 expression for the indicated conditions. (D) Single-cell analysis of RhoB activation by time-lapse microscopy. Plot of the ratio of cellular mean fluorescence intensity (MFI) to basal MFI measured at T0 (MFI_T0_) for serum-starved and serum-stimulated cells [*n*=20 cells from four (serum stimulation) and five (serum starvation) independent experiments; mean±s.e.m.]. An enlargement is shown on the right for serum-stimulated condition (0–240 min).

We engineered a single plasmid that expresses both the GTPase and the GTPase-binding domain of its effector from a bidirectional promoter (bi-promoter). This allows simultaneous expression of the two transgenes in MRC5-SV cells that stably expresses the GFP1-9 fragment and the anti-GFP VHH domain (Fig. 3A; Fig. S4A). We applied this system to the detection of RhoB active form, since this GTPase requires low cellular expression levels to preserve cell physiology. Lentiviral particles for GFP10-RhoB and RBD-GFP11 were used to transduce a model of MRC5-GFP1-9 cells that were sorted for enrichment in GFP-positive cells. Split-GFP complementation was tested prior to and after transduction of the GFP enhancer in a triSFP RhoB–RBD activation cell line model. We observed a doxycycline dose–response expression from the bi-promoter system with a 4- to 6-fold improvement in cell fluorescence compared to the initial model in the absence of the nanobody, while basal doxycycline-independent expression was undetectable in the RhoB activation reporter cell line (Fig. 3B).

RhoB activation was also assessed after stimulation, in conditions where cells were grown in serum-starved conditions, then stimulated or not with 10% FBS for 4 h before analysis of cell fluorescence by flow cytometry. Stimulation of serum-starved reporter cells with serum led to a 1.5-fold increase in the percentage of fluorescent cells compared to control non-stimulated cells (Fig. 3C). The percentage of fluorescent cells in untreated conditions was unchanged at between no Dox (T0) and at 4 h Dox treatment, indicating that there was no detectable variation in RhoB activation during that timeframe. Our results indicate that there is a high percentage of fluorescent cells in basal conditions, which reflects the strong activation of RhoB upon expression, consistent with *rhob* being a gene that immediately responds to growth factors and cellular stress (Canguilhem et al., 2005; Gampel and Mellor, 2002). To analyze the increase in fluorescence upon stimulation, we performed time-lapse experiments in starved RhoB biosensor cells maintained in steady-state conditions or in cells stimulated with 10% FBS. Analysis of single-cell fluorescence indicated an increase in RhoB active form at 20 min post stimulation, which accumulated to reach a 1.4-fold increase in the average mean fluorescence intensity of individual cells relative to unstimulated cells after 4 h (Fig. 3D, inset). In contrast, starved cells showed no significant variation of the fluorescence intensity during the image acquisition time. This result correlates closely with the increase in fluorescence observed with FACS measurements on a global population of cells (Fig. 3C).

### Analysis of Rho activity inhibition

**Fig. 4.**
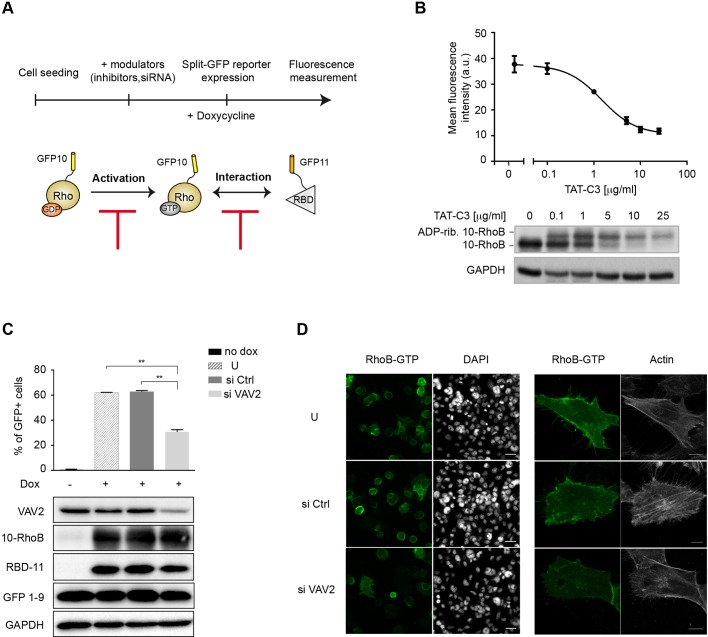
**Analysis of modulation of RhoB activity.** (A) Experimental workflow used to evaluate inhibitors of Rho activation by using the triSFP Rho reporter system; inhibitors or siRNAs were added prior to split-GFP expression. A decrease in split-GFP fluorescence will report either a decrease of the amount of the active GTP-Rho form or the inhibition of the Rho–RBD interaction. (B) Dose–response inhibition of the RhoB reporter with TAT-C3 exoenzyme (0.1 to 25 µg/ml). The mean fluorescence intensity was analyzed by flow cytometry. Results are mean±s.e.m.; *n*=3. As shown in the blot underneath the graph, an analysis of RhoB expression (anti-GFP10) confirmed the ADP-ribosylation (rib.) of RhoB that induces a shift of the RhoB band. (C) Percentage of GFP fluorescent cells for RhoB reporter cells left untransfected (U) or transfected with scrambled siRNA (siCtrl) or siRNA targeting the RhoGEF VAV2 (siVAV2). Results are mean±s.e.m.; *n*=3 independent experiments. **P<0.01 (Student's *t*-test). Expression of VAV2 and triSFP components is shown below (anti-GFP10 and anti-GFP11 antibodies; GFP1-9, antibody against full-length GFP). (D) Representative images of untransfected (U), siCtrl and siVAV2-transfected cells visualized by fluorescence microscopy. Left, wide-field images of cells expressing active RhoB (FITC channel); DAPI nuclear staining. Scale bars: 50 µm. Right, representative confocal images of RhoB-GTP and actin labeling (Phalloidin–Alexa-Fluor-594). Scale bars: 10 µm.

Small GTPase activation is directly regulated by GEFs that catalyze GDP exchange with intracellular GTP (Rossman et al., 2005). To explore the sensitivity of our above assay to report an inhibition of Rho activation signaling, we prevented split-GFP reporter complementation by adding modulators before split-GFP reporter expression (Fig. 4A). We first tested the effect of exoenzyme C3 transferase, which ADP-ribosylates Rho on Asn41 at the edge of the switch-I region and consequently inhibits nucleotide exchange catalyzed by GEFs (Sehr et al., 1998). A cell permeable version of the C3 exoenzyme (TAT-C3) (Sahai and Olson, 2006) was added prior to reporter expression, which prevented, in a dose-dependent manner, the triSFP RhoB activation fluorescence with a complete inhibition above 10 µg/ml of purified TAT-C3 exoenzyme (Fig. 4B). Analysis of the corresponding cell extracts indicated that the loss of fluorescence correlated with the incremental formation of ADP-ribosylated RhoB, thus confirming that the biosensor model relies on early Rho activation events mediated by GEFs. At high concentrations of Rho inhibitors (>5 µg/ml), the short-lived RhoB GTPase is preferentially degraded in its ADP-ribosylated form (Fig. 4B). Furthermore, to evaluate whether the RhoB-split-GFP reporter can be used as a GEF activity readout, we evaluated the downregulation of VAV2, a major RhoGEF known to activate RhoB and other GTPases upon activation of growth factor receptors (Gampel and Mellor, 2002; Liu and Burridge, 2000; Thalappilly et al., 2010). Inhibition of VAV2 expression through RNAi resulted in a 2-fold decrease in the percentage of GFP-positive cells, which in turn correlated with a significant decrease in the GFP fluorescence of the global population (Fig. 4C) and in cells expressing GFP10-RhoB and RBD-GFP11 (Fig. S4B). These results highlight the marked contribution of VAV2 to RhoB activation (Fig. 4C), and suggest that it may participate in basal RhoB activation in conditions of overexpression. Fluorescence imaging of the same treated cells confirmed the decrease in split-GFP-positive cells (Fig. 4D, left). This correlated with a dimer fluorescence of intracellular and membrane regions, and with the loss of actin stress fibers, corroborating loss of Rho functions (Fig. 4D, right). Taken together, our data further validate the triSFP RhoB activation model as a robust tool to analyze inhibition of Rho activity linked to intrinsic interactions mechanisms between Rho and its upstream regulators.

### Monitoring modulation of Rho activation using high-content analysis

**Fig. 5.**
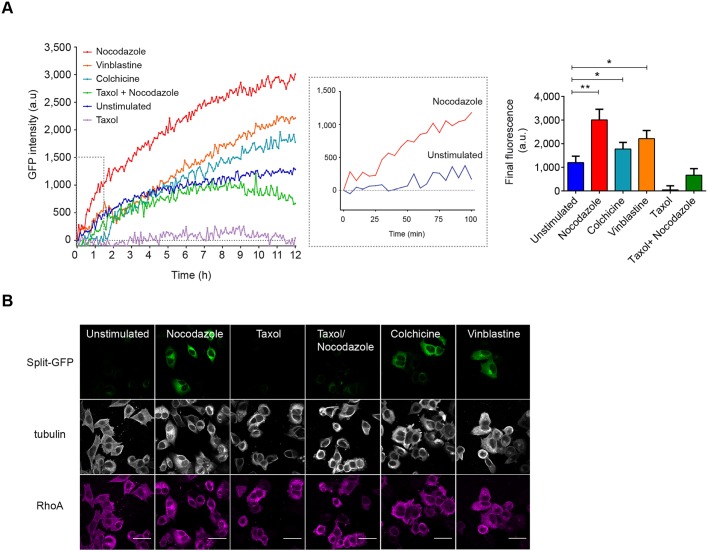
**RhoA activation with microtubule drugs.** (A) A RhoA activation reporter cell line was engineered to analyze RhoA activation upon treatment with microtubule poisons in a 96-well format. GFP intensity kinetic curves show modulation of RhoA activation upon treatment with indicated compounds (mean of three independent acquisitions). Middle graph, nocodazole shows the strongest induction of GFP fluorescence (*y*-axis shows arbitrary units), highlighting the increase of split-GFP fluorescence early after stimulation. The bar graph on the right shows the final GFP fluorescence value for each condition. Results are mean±s.e.m.; *n*=3, *P<0.05, **P<0.01 (Student's *t*-test). (B) Representative confocal images of split-GFP fluorescence (FITC channel), α-tubulin (gray) and RhoA expression (anti-GFP10, magenta) for the indicated treatments. Scale bars: 40 µm.

With the goal of miniaturizing our method and facilitating the screening of multiple conditions, we adapted the triSFP Rho assay to 96-well plate format analysis. We also transposed the split-GFP reporter system to RhoA, for which the regulation mechanisms have been more extensively studied. The lentiviral vector encoding both the GFP10-RhoA and RBD-GFP11 fusions was transduced in MRC5_GFP1-9_VHH cells and enriched based on split-GFP fluorescence. As expected, the cell model displayed doxycycline-regulated expression of RhoA and RBD chimera, and showed partial inhibition of RhoA activity upon treatment with 10 µg/ml TAT-C3 peptide (Fig. S5). To exemplify our approach, we focused on several cytoskeleton-destabilizing drugs that have been shown to deregulate Rho activity upon microtubule collapse. The proposed mechanism of RhoA activation is the release of GEF-H1 (Krendel et al., 2002), which controls Rho GTPase activation in coupling with microtubule dynamics to promote actin stress fiber formation and cell contractility (Chang et al., 2008). To evaluate whether such mechanisms could be detected with our assay, we tested several microtubule-destabilizing agents (nocodazole, colchicine and vinblastine) that induce microtubule depolymerization, and evaluated the effect of paclitaxel (Taxol), which promotes microtubule stabilization (Jordan and Wilson, 2004). triSFP RhoA activation reporter cells were maintained without serum for 24 h, then fluorescence was monitored with a fluorescence plate reader in the presence of the various agents for 12 h. Analysis of the mean velocity of GFP fluorescence formation indicated a 3-fold increase in RhoA activation in nocodazole-treated condition compared to unstimulated cells, with vinblastine and colchicine treatment causing a 2-fold increase (Fig. 5A). No fluorescence increase was observed upon Taxol treatment and in nocodazole-stimulated cells pre-treated with 10 µM Taxol. The lower fluorescence kinetic curve observed for Taxol reflects an apparent cellular toxicity of this compound that prevented basal RhoA activation (as observed in unstimulated cells) (Fig. 5A). Imaging of one representative field of the corresponding well stained for α-tubulin and for GFP10 confirmed that the drugs had the expected activity on tubulin cytoskeleton and highlighted bright GFP fluorescent cells in conditions with unpolymerized tubulin (Fig. 5B). In contrast, Taxol-treated or pre-treated cells showed background fluorescence that was similar to that of unstimulated cells with GFP10-RhoA expression (anti-GFP10 staining). Taken together, our results indicate that the assay is able to report a described RhoA activation mechanism, which is regulated through microtubule depolymerization.

**Fig. 6.**
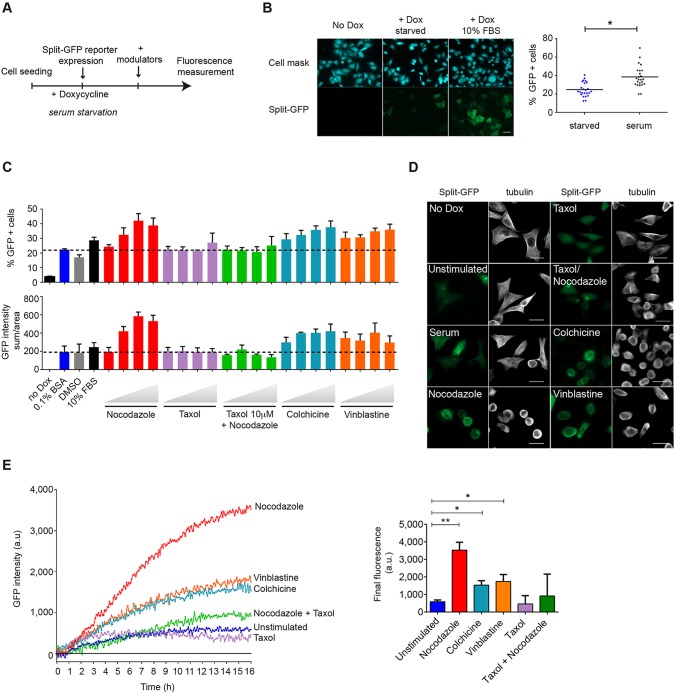
**High-content analysis of RhoB activation.** (A) Protocol used to evaluate RhoB modulators; the split-GFP reporter was expressed in serum-starved conditions prior to stimulation. (B) RhoB activation reporter cells were stained with a blue cell mask for quantification of the percentage of GFP-positive cells on a high-content Operetta imaging^®^ system. The threshold for quantifying GFP-positive cells is set relative to that in uninduced conditions (no Dox). The graph shows an analysis of eight single measurements in serum-starved (+Dox, starved) and serum-stimulated (+Dox, 10% FBS) conditions. Results are mean±s.e.m.; *n*=3 independent experiments. *P<0.05 (paired Student's *t*-test). (C) Quantification of RhoB activation after treatment with the indicated compounds. Experiments were performed to analyze the percentage of GFP-positive cells or the sum of GFP intensities on the cell surface area. Controls: uninduced (no Dox), induced starved-conditions (0.1% BSA) or 0.1% BSA with DMSO (DMSO) or serum (10% FBS). Compounds: nocodazole (0.2, 1, 5, 10 µM), Taxol (1, 2, 5, 10 µM), 10 µM Taxol with 30 min treatment prior to nocodazole stimulation (0.2, 1, 5, 10 µM), colchicine (0.1, 1, 10, 25 µM), vinblastine (50 and 100 nM, 1 and 5 µM). Results are mean±s.e.m. of three independent experiments. For each experiment, data are means of eight replicates for no Dox, serum and starved control conditions, and duplicates for the other conditions. (D) Representative microscopy images of the corresponding high-content plate analysis. Split-GFP fluorescence is shown by the FITC channel and α-tubulin staining is in gray. Scale bars: 40 µm. (E) Fluorescence kinetic curves of RhoB activation following stimulation with 5 µM nocodazole, 10 µM Taxol, 10 µM Taxol+5 µM nocodazole, 1 µM colchicine and 0.1 µM vinblastine. Final GFP fluorescence values for the indicated conditions are shown on the right. Results are mean±s.e.m.; *n*=3 independent experiments. *P<0.05; **P<0.01 (Student's *t*-test).

As little is known about RhoB activation in this context, we tested how these agents act on the modulation of RhoB activity. The triSFP RhoB activation reporter cell line was further cell-sorted by flow cytometry based on split-GFP complementation fluorescence in serum-cultured conditions in order to obtain a homogenous population of cells that express both the RhoB and RBD chimera (Fig. S4C). In order to sensitively detect single-cell changes in GFP fluorescence, we adapted the assay to single-cell analysis by using an automated high-content screening microscope. The triSFP RhoB activation reporter was induced in serum-starved conditions before stimulation with the drugs (Fig. 6A). We first tested the efficiency of the system to report serum-induced RhoB activation by quantifying the percentage of GFP-positive cells by comparison to a cell mask revealing all cells, which reproduced serum-induced RhoB activation in this format (Fig. 6B). We next calculated the Z-factor to determine whether the assay is suitable for high-content screening (Zhang et al., 1999). Our results revealed Z-factor values of 0.5 in the context of 50% inhibition with TAT-C3 exoenzyme (1 µg/ml TAT-C3) and of 0.8 in the context of 75% inhibition (5 µg/ml TAT-C3) (Fig. S6A), indicating that the assay achieves acceptable performance for screening applications. To take advantage of the throughput, various doses of the microtubule-targeting drugs were tested in an overnight stimulation protocol and quantification of the split-GFP fluorescence was performed after cell fixation. Single-cell GFP fluorescence was calibrated on the serum-starved cells' basal fluorescence [0.1% bovine serum albumin (BSA)]. We observed a linear increase in the percentage of GFP-positive cells with the concentration of microtubule-depolymerizing drugs, which indicates the dose-dependent quantitative response of the reporter to RhoB activation (Fig. 6C). No activation was detected with Taxol or with the Taxol and nocodazole combination, as expected. As was seen for RhoA, RhoB displayed a preferential activation after nocodazole treatment, followed by vinblastine and colchicine treatment. The lower activity of these agents correlated with the results of GST-RBD pulldowns on endogenous RhoB from HeLa cells extracts (Fig. S6B). GFP fluorescence of cells inversely correlated with the amount of α-tubulin bundles (Fig. 6D). We noticed a higher fluorescence background of RhoB in starved cells and after treatment with polymerizers when compared to the background with RhoA (see Fig. 5B), which indicates that a basal RhoB activation response is induced due to its expression. To compare the RhoB activation profile upon treatment with these drugs, we analyzed, on a fluorescence plate reader, the time course of the appearance of active RhoB in response to these stimuli. In accordance with the high-content imaging results, we observed a higher induction of RhoB activation with nocodazole than with colchicine and vinblastine, and no induction of RhoB activation in Taxol-treated cells (Fig. 6E). Final fluorescence values indicated a stronger response for RhoB to microtubule-depolymerizing drugs than seen with RhoA, with a 6-fold increase in fluorescence for nocodazole and a 3-fold increase for colchicine and vinblastine relative to that in unstimulated conditions. These results highlight a conserved specificity of these drugs towards RhoA or RhoB activation while the two Rho homologs differ in their activation profile. These results demonstrate that such analysis could be broadened to other GTPase-activating systems to evaluate their sensitivity to a drug and determine the relative drug efficacy towards their active state.

## DISCUSSION

To investigate the modulation of Rho activation in live cells, we developed a versatile fluorescent reporter system for small GTPase activation state based on a tripartite split-GFP assay. We demonstrated that this tagging system, which involves β-strands of the GFP that are 20 amino acids long, does not interfere with the activity and subcellular localization of Rho and Ras mutants (see Fig. 1B). The method provides a practical, direct fluorescent readout of the GTPase active form in live cells, which can be either analyzed by flow cytometry or by fluorescence microscopy, to obtain quantitative measurements of the reporter fluorescence under different treatment conditions. We validated the adequate reporter response relative to the GTPase activity by analysis of interactions between active and inactive forms of small GTPases with their cognate effector domain. Our results indicate very low background fluorescence levels with dominant-negative variants, and a 5- to 12-fold increase in fluorescence levels for the constitutively activated Rho and Ras mutants (Fig. 1D). This strong dynamic range is likely related to the irreversibility of the split-GFP association, for which GFP fluorescence accumulates over time. By stabilizing transient events of GTPase activation, the split-GFP tagging scheme offers a new alternative method that provides a wide dynamic range and time detection window to study the function of small GTPases in various detection formats.

To study RhoB activation in single cells, we developed an inducible stable cell-based model that integrates regulated expression of the reporter together with an anti-GFP VHH intrabody (Kirchhofer et al., 2010) that enhances split-GFP fluorescence. The nanobody-enhancing properties on complemented split-GFP might involve the stabilization of interactions of the three-body split-GFP fragments after complementation, which might, in turn, facilitate chromophore maturation. Our finding that the anti-GFP intrabody does not bind to GFP1-9 in the absence of interaction enables the stable expression of the VHH without interfering with the tripartite split-GFP association. This strategy provides a higher sensitivity of the triSFP assay in the context of low expression, as for RhoB, and in high-content assays of mammalian cells (Figs 3 and 6). Such a cellular model will be particularly well suited for evaluating Rho mutations, inhibitors and siRNA downregulation of factors that may affect the GTPase activation state. Accordingly, we have shown that the assay could detect downregulation of VAV2-induced activation of RhoB (Fig. 4C). VAV2 is known to activate other GTPases, such as RhoA, Rac1 and Cdc42 (Abe et al., 2000), and its downregulation may therefore directly affect RhoB activation or indirectly affect it via the modulation of other GTPases that may cross-talk with RhoB. Such models could be extended to other small GTPases of the Ras superfamily to interrogate GEFs activity in a focused study.

We demonstrated that we could monitor the response of Rho activation to various microtubule poisons in RhoA and RhoB triSFP reporter cell models. For both GTPases, this mechanism is mediated by GEF-H1 release upon microtubule depolymerization (Arnette et al., 2016; Chang et al., 2008). The higher efficiency of nocodazole relative to colchicine and vinblastine towards Rho activation (either determined with the split-GFP assay or on endogenous proteins) may be linked to the mode of action of these drugs on microtubule disassembly, which might affect GEF release. The assay reliably reported inhibition in Taxol-pretreated nocodazole-stimulated cells, which indicates its robustness and sensitivity in detecting a combination of multiple drugs. Likely, such a Rho activation model could be useful to screen for compounds that affect tubulin polymerization in cell-based assays.

The building model strategy described in this study may be transferred to other binding domains whose activity depends on conformational changes or interactions with regulatory and effector proteins. Our results indicate that the assay is readily adaptable to other GTPase and effector systems, and other interacting protein–protein pairs to perform a robust evaluation of the modulation of the interaction by small molecules in cell-based assays. The robustness of such a system for high-content analysis relies on the strong specificity of the tripartite split-GFP complementation so that its does not display unspecific binding leading to a false signal, and on the accumulation of the split-GFP signal so that its does not limit the timing of the assay, in contrast to dynamic probes. Indeed FRET probes allowed us to monitor the real-time GTPase activation kinetics while the irreversibility of the split-GFP assay enabled us to capture transient events of activation or inactivation, creating a stable signal to identify compounds affecting transient GTPase activation.

Our system can also be used to analyze the specificity of interaction of GTPases and their signaling partners (regulators and effectors). In these assays, transient expression and localization of the individual protein domains can be assessed robustly by the dual labeling of GFP10- and GFP11-tagged proteins with specific anti-tag antibodies. This can be combined with the analysis of their interaction with split-GFP complementation. The modularity of the split-GFP tagging scheme facilitates the transposition of the assay to other proteins of the Ras superfamily of small GTPases in order to monitor their activation in response to various stimuli independently of their effect on expression. These results open the door for the identification of specific modulators of small GTPase activation such as GEFs and related domains, and to monitor changes of their active state after stimulation, inhibition with small molecule agents or downregulation of upstream signaling. We anticipate that such a model will find applications in various signaling modules to evaluate the effect of chemical and pharmacological compounds on their activity.

## MATERIALS AND METHODS

### Cell lines and reagents

Human embryonic kidney (HEK) 293 cells and MRC5-SV (immortalized normal pulmonary human fibroblasts) cells were cultured in Dulbecco's modified Eagle's medium (DMEM) and 10% (v/v) fetal bovine serum (FBS) (Lonza, Basel, Switzerland) and routinely checked for mycoplasma contamination. MRC5-SV cells medium was supplemented with 100 µg/ml Normocin (InvivoGen). Transfection of plasmids or siRNA was performed using jetPRIME (Polyplus-transfection, France) according to manufacturer's instructions. The siRNA-targeting sequence for RNA interference of VAV2 (siVAV2) was the sequence 5-GGACAUCAACUUCCGGCCGdTdT-3 and its corresponding complement 5-CGGCCGGAAGUUGAUGUCCdTdT-3 (Gampel and Mellor, 2002), which were synthesized and annealed by Eurogentec (Seraing, Belgium). siRNA duplex negative control SR-CL000-005 (Eurogentec) was used as control siRNA (siCtrl). C3 exoenzyme coupled to permeant peptide TAT was produced and purified in our laboratory as previously described (Sahai and Olson, 2006). Microtubule poisons were purchased at the following manufacturers: nocodazole (Sigma), paclitaxel (Selleckchem), colchicine (Sigma), vinblastine (Sigma).

### Vector design

RhoA, RhoB and HRas mutants were generated by site-directed mutagenesis and cloned into the BspeI:XbaI sites of pcDNA_GFP10-Nter fusion vector. The RBD of Rhotekin and RsBD of RAF-1 were amplified by PCR from pGST-RBD pGEX (Addgene #15247) and Raf-1 GST-RBD 1-149 pGEX (Addgene #13338) and inserted into NotI:ClaI cloning sites of pcDNA_GFP11-Cter fusion vector. The VHH G4 anti-GFP cDNA (gift from Aurélien Olichon, Cancer Research Center of Toulouse, France) was amplified by PCR and cloned into NheI/BamHI restriction sites of the lentiviral vector pTRIP CMV (gift from Loïc VandenBerghe, ‘Vectoul’ platform, Cancer Research Center of Toulouse, France). Human NES immunodeficiency virus Rev (LPPLERLTL) and KRas-4B-CAAX (KMSKDGKKKKKKSKTKCVIM) targeting sequences were generated by PCR assembly by using synthetic oligonucleotides, and were cloned into XhoI/BamHI sites of the pCMV_GFP1-9 vector. The tet-on inducible bidirectional promoter lentiviral vector (Lv. pTRIP TRE-BI GFP10-Rho/RBD-GFP11) was generated by subcloning the RBD-GFP11 cassette from a pTRE tight BI vector (Clontech) into the MLuI site of an HIV-1-based lentiviral pTrip vector carrying a tetracycline response element (TRE) (BIVIC platform, IFR 150, CHU Rangueil, Toulouse, France) (see Fig. S4A). Plasmids designed for the study are listed in Table S1.

### Generation of Rho activation reporter cell lines

Lentiviral vector particles for GFP1-9, anti-GFP VHH and pTRIP TRE-BI GFP10-Rho/RBD-GFP11 vectors were produced by co-transfection of lentiviral vectors with the p8.91 and pLvVSVg packaging vectors in HEK293FT cells. Viral supernatants were harvested 48 h after transfection, and filtered through 0.45 μm filter units (Millipore). The virus preparations were aliquoted and frozen at −80°C. Sequential transductions with lentiviruses for GFP1-9 and GFP10-Rho/RBD-GFP11 were performed at a multiplicity of infection (MOI) of 2 in MRC5-SV rtTA cells seeded in six-well plates. After recovery, cells were sorted by flow cytometry based on GFP fluorescence after induction with 1 µg/ml doxycycline for 24 h. Transduction of the anti-GFP VHH lentivirus was subsequently performed on the optimized cell line and in the parental MRC5-SV_GFP1-9 cells in the same experimental conditions. For high-content imaging experiments, an additional cell-sorting step was performed to enrich in cells that express homogenous levels of the reporter (see Fig. S4C).

### Western blotting

Whole-cell extracts were obtained with a lysis buffer (1% SDS, 10 mM Tris-HCl pH 7.4, protease inhibitor cocktail and Halt phosphatase inhibitor cocktail 3; Sigma-Aldrich). After viscosity reduction of the cell lysate by brief sonication, the proteins were separated by SDS-PAGE and immunoblotted with the indicated antibodies (see Table S2). Immunoblotting was revealed by chemiluminescence using the ChemiDoc MP system (Bio-Rad, Hercules).

### Flow cytometry analysis and double immunostaining protocol

For transient expression of GTPases and the domain of their effector, 4×10^5^ HEK_1-9 cells were seeded in six-well plates and transfected at a ratio 1:1 of the GFP10 and GFP11 fusion vectors (1 µg) with mock plasmid (1 µg). One control well was transfected with 10-Z-11 encoding plasmid (1 µg+1 µg mock plasmid) and one condition was left untransfected. For characterization of the anti-GFP VHH booster, MRC5_GFP1-9 and MRC5_GFP1-9-VHH cells were seeded in six-well plates at a density of 3.5×10^5^ cells per well and transfected with 2 µg of DNA (1:1 ratio of GFP10/GFP11 fusions, or 1:1 ratio of mock plasmid with 10-Z-11 and 10-R-11, or eGFP plasmid). At 20–24 h after transfection, cells were trypsinized, resuspended in 2% FBS, PBS then transferred in 96-well conical bottom plates for intracellular staining steps. Cells were fixed with 3.7% paraformaldehyde (PFA) for 10 min and permeabilized with 0.1% Triton X-100 in 1% BSA in PBS buffer. Blocking was performed with 8% BSA in PBS for 1 h before adding primary antibodies and incubation. For dual labeling, primary anti-GFP10 rabbit and anti-GFP11 mouse antibodies were added at 1:1000 dilution for 2 h, followed by secondary labeling (1:800 dilution, 45 min) with Pacific Blue anti-rabbit and Alexa-594 anti-mouse IgGs. GFP fluorescence was determined for the gating region corresponding to double GFP10+ and GFP11+ labeling (defined on double immunostaining of untransfected cells) on a MACS Quant cytometer. The percentage of GFP-positive cells in the global population and GFP intensity in the gated region was analyzed with Kaluza® and FlowJo® software.

### Immunofluorescence and microscopy analysis

Cells on coverslips or 96-well plates were fixed with 3.7% PFA and permeabilized with 0.1% Triton X-100 in PBS buffer. For double GFP10 and GFP11 immunofluorescence, similar antibodies and incubation times were used as for intracellular staining analysis. Coverslips were mounted using Mowiol (Calbiochem, EMD Millipore) supplemented with DAPI for nuclei staining. Individual cells were imaged with a LSM 780 (Zeiss, Oberkochen, Germany) confocal laser scanning microscope using a 488 Argon laser with a 490–553 nm emission filter (GFP). Alexa Fluor 594 and DAPI labeling were acquired with an argon laser (543 nm) and 405 UV diode lasers, respectively, using 20× and 63×1.4 NA oil immersion objectives. Image analysis was performed with ImageJ® software. For 96-well plate staining, the cells were fixed with 3.7% PFA, permeabilized with 0.1% Triton X-100 in PBS buffer and stained with corresponding antibodies (see Table S1).

For time-lapse imaging of living cells, cells were grown on µ-Slide 8-well ibiTreat chambered coverslips (Ibidi, Biovalley). Acquisition was performed with a LSM 780 confocal microscope using a complete Pecon-Zeiss incubation system for a temperature (37°C) and CO_2_ (5%) controlled environment. The same laser intensity, gain, scan-speed and zoom were used for all experiments. For quantification, ten *z*-stack confocal images were acquired (every 60 nm) at the indicated time intervals and duration. The *z*-stack of each cell was transformed into *z* projection by summing the ten *z*-stacks for all the time frames. The same threshold was then applied for all analyzed images before plotting the *z*-axis profile of mean fluorescence intensity along the time.

### Analysis of Rho inhibition

For characterization of the Rho active reporter model, cells were seeded at a density of 250,000 cells per well in six-well plates. Split-GFP reporter expression was induced with 2.5 µg/ml doxycycline in serum-free DMEM supplemented with 0.1% (mol/vol) fatty acid-free BSA for 24 h. Stimulation was performed with enriched (10% FBS) fresh medium. For inhibition experiments with TAT-C3 peptide, 24 h after cell seeding, the medium was replaced by a doxycycline- and TAT-C3-containing medium for 48 h. GFP fluorescence was analyzed by flow cytometry (percentage of GFP-positive cells). In siRNA downregulation experiments, the cells were cultured for 24 h then transfected with the indicated siRNA. After 72 h, the split-GFP reporter expression was induced with doxycycline-containing medium for 24 h. Cells were processed for a double intracellular staining protocol with anti-GFP10 and anti-GFP11 antibodies before flow cytometry analysis.

### High-content analysis of Rho activation

RhoB and/or RhoA triSFP reporter cells were seeded in 96-well plates (Greiner) at a density of 8000 cells/well (kinetic measurement) and 4000 cells/well (final point measurements). After 24 h, the medium was changed to starvation medium (DMEM, 0.1% BSA) supplemented with doxycycline (1 µg/ml or 0.25 µg/ml for kinetic and final point experiences, respectively).

Before live measurement, the medium was replaced after PBS washing with DMEM/HEPES-buffered medium without Phenol Red (Sigma, D6434) and drugs were added at the indicated concentrations. In the Taxol and nocodazole combined treatment condition, cells were pre-treated with 10 µM Taxol for 30 min before nocodazole treatment. Acquisition was performed on a Synergy2 fluorescence plate reader (BioTek) in a 37°C controlled chamber with a 488/525 nm filter for 16 h. Data was analyzed with Gen5 software (Biotek); the background GFP fluorescence from non-induced control cells was subtracted from the obtained GFP fluorescence (six replicates) and normalized to the initial fluorescence value. Final fluorescence was determined by subtracting corrected sample fluorescence values (three replicates) obtained from the DMSO mock sample (three replicates).

For final point measurement experiments, cells were fixed with 3.7% PFA after 16 h of treatment with the microtubule drugs, then stained for tubulin, and a cell mask was obtained with HCS CellMaskTM Blue Stain (ThermoFisher Scientific) according to the supplier's instructions. Quantitative image acquisition was performed on an Operetta high-content imaging system (Perkin Elmer) with a 20× objective lens in the 488/525 nm (GFP) and 360/405 nm (cell mask) channels. Analysis was performed with Harmony® software on an average of 1500 cells/well. The number of objects and the sum of cell area was determined from the cell mask staining. The percentage of GFP cells was calculated as: percentage GFP cells=(number of GFP-positive cells/number of all objects)×100, where GFP-positive cells are defined by cells following this criteria: mean of fluorescence intensity (MFI) of the object>mean of MFI in the control wells without doxycycline. GFP intensity sum/area is defined as the (sum of GFP intensity sum) of each object in the well/(sum of object area sum) of each object in the well.

### Quantification and statistical analysis

Data analysis was performed by using GraphPad Prism software v.6. All data are presented as means±standard error of the mean (s.e.m.) of the indicated number of independent experiments. The statistical significance of differences between two groups was evaluated with a Student's *t*-test. For multiple two group comparisons, the Holm–Sidak method with α=0.05 was applied.

## Supplementary Material

Supplementary information
