# Supplementary material for: High-content tripartite split-GFP cell-based assays to screen for modulators of small GTPase activation
Source: J Cell Sci. 2018 Jan 1;131(1):jcs210419. doi: 10.1242/jcs.210419 (PMC5818064; doi:10.1242/jcs.210419)
Supplement: Supplementary information [file joces-131-210419-s1.pdf]

Supplementary information

High-content tripartite split-GFP cell-based assays to screen for modulators of small GTPase activation

Faten Koraïchi[1,2], Rémi Gence[1,2], Catherine Bouchenot[1,2], Sarah Grosjean[1,2], Isabelle Lajoie-Mazenc[1,2], Gilles Favre[1,2,*] & Stéphanie Cabantous[1,2, 3*].

This file includes:

Supplementary Figure S1, related to Figure 1. Characterization of split-GFP chimera and antibodies used in the study.

Supplementary Figure S2, related to Figure 1. FACS analysis controls for triSFP assays on Rho and Ras mutants.

Supplementary Figure S3, related to Figure 2. Control of split-GFP chimera expression on triSFP quantification with VHH GFP booster.

Supplementary Figure S4, related to Figure 3 and 4. Design and characterization of the RhoB activation triSFP reporter cell model.

Supplementary Figure S5, related to Figure 5. Characterization of the RhoA activation triSFP cell model.

Supplementary Figure S6, related to Figure 6. High-content analysis of RhoB activation.

Supplementary Table S1: List of plasmids used in the study

Supplementary Table S2: List of antibodies used in the study.

Journal of Cell Science • Supplementary information

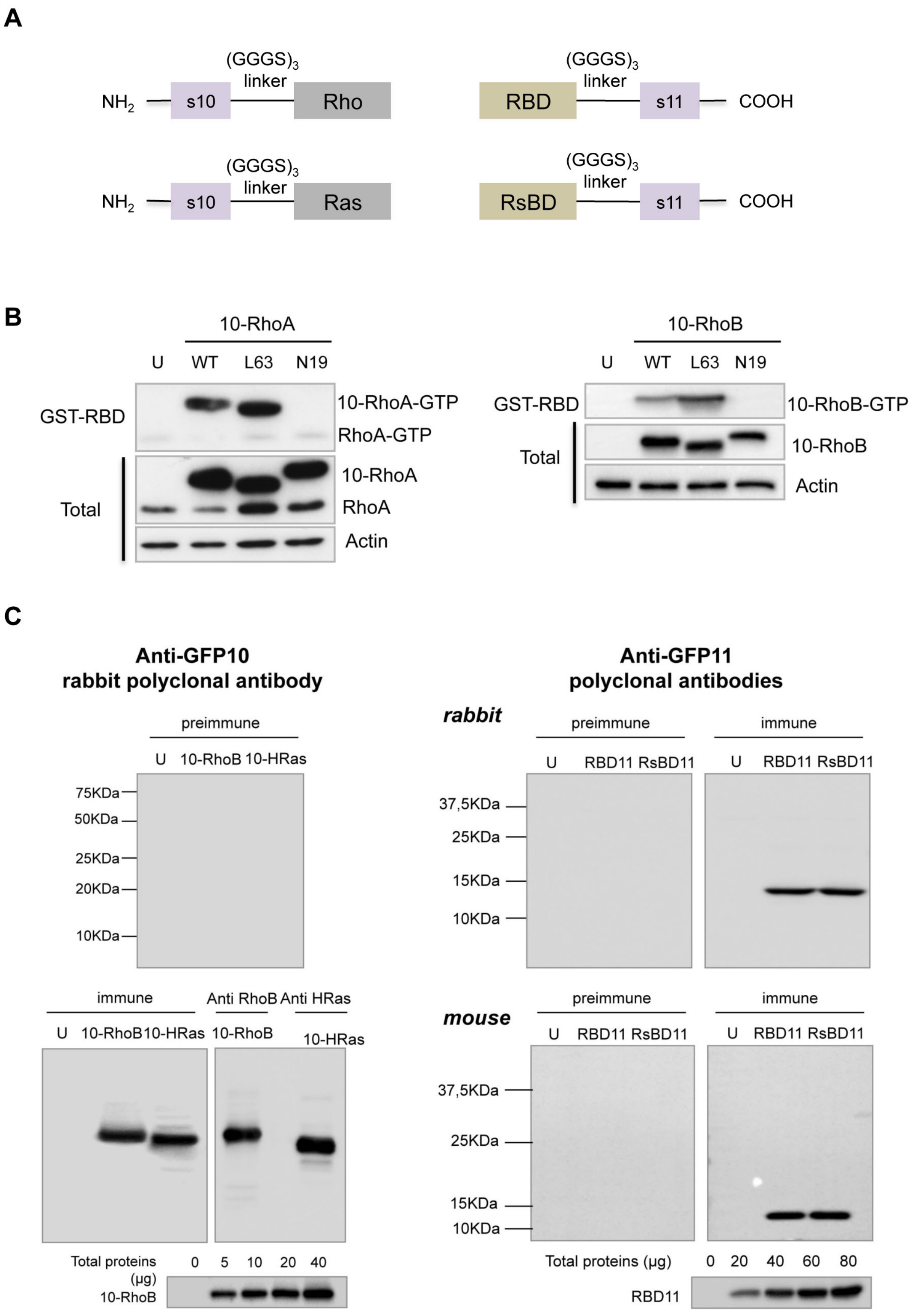

Figure S1, related to Figure 1. Characterization of split-GFP chimera and antibodies used in the study. (A) Split-GFP tagging scheme for GTPases and effector domains. The

Journal of Cell Science • Supplementary information

GFP10 tag (s10) was fused to the N-terminus of GTPase, leaving their C-terminus free for translational modifications. Rho and Ras binding domains (RBD/RsBD) were fused C-terminaly with GFP11 (s11) to preserve the antiparallel topology of β10-β11 in the reconstituted GFP complex. Flexibility between fusion protein and the tag is provided by a 12 mer (GGGS) linker. (**B)** Binding of GFP10-Rho chimera on GST-RBD beads was tested on cell extracts of HEK 293 cells either untransfected (U) or transfected with GFP10 fusions for wild-type (WT), constitutively active Q63L mutants (L63) or dominant negative T19N mutants (N19) of RhoA or RhoB. Representative western blot analysis of the active fraction of RhoA or RhoB captured on GST-RBD beads is shown in the upper gels of each panel. The lower gels show western blot analysis of the total cell lysates collected prior to the incubation with GST-RBD beads and used as a loading control. Samples were blotted with anti-RhoA and anti-RhoB commercial antibodies, revealing both GFP10-Rho and endogenous Rho proteins. (**C)** Specificity of the anti-GFP10 and anti-GFP11 immune sera for Western Blot analysis was tested on cell extracts from untransfected HEK 293 cells (U) or transfected with GFP10 and GFP11 fusions proteins for 24 h. For each antibody, the corresponding preimmune serum (collected prior to animal immunization) was used as negative control. (**left**) Western blot analysis shows the detection of preimmune and immune sera (dilution 1:2000) of cell extracts expressing GFP10-RhoB or GFP10-HRas and were compared to commercial anti-RhoB and anti-HRas antibodies. (**right**) For both rabbit and mouse species, anti-GFP11 immune serum was validated on cell extracts from HEK 293 cells either untransfected (U) or transfected with RBD-GFP11 or RsBD-GFP11 for 24h. The immune and preimmune sera were used at a 1:2000 dilution. Sensitivity of rabbit anti-GFP10 or anti-GFP11 antibodies were tested on loaded protein quantity in WB analysis. GFP11 sequence used for immunization corresponds to GFP11 M4 from tripartite SFP; anti-GFP11 antibodies described here do not recognize bipartite GFP11 M2.

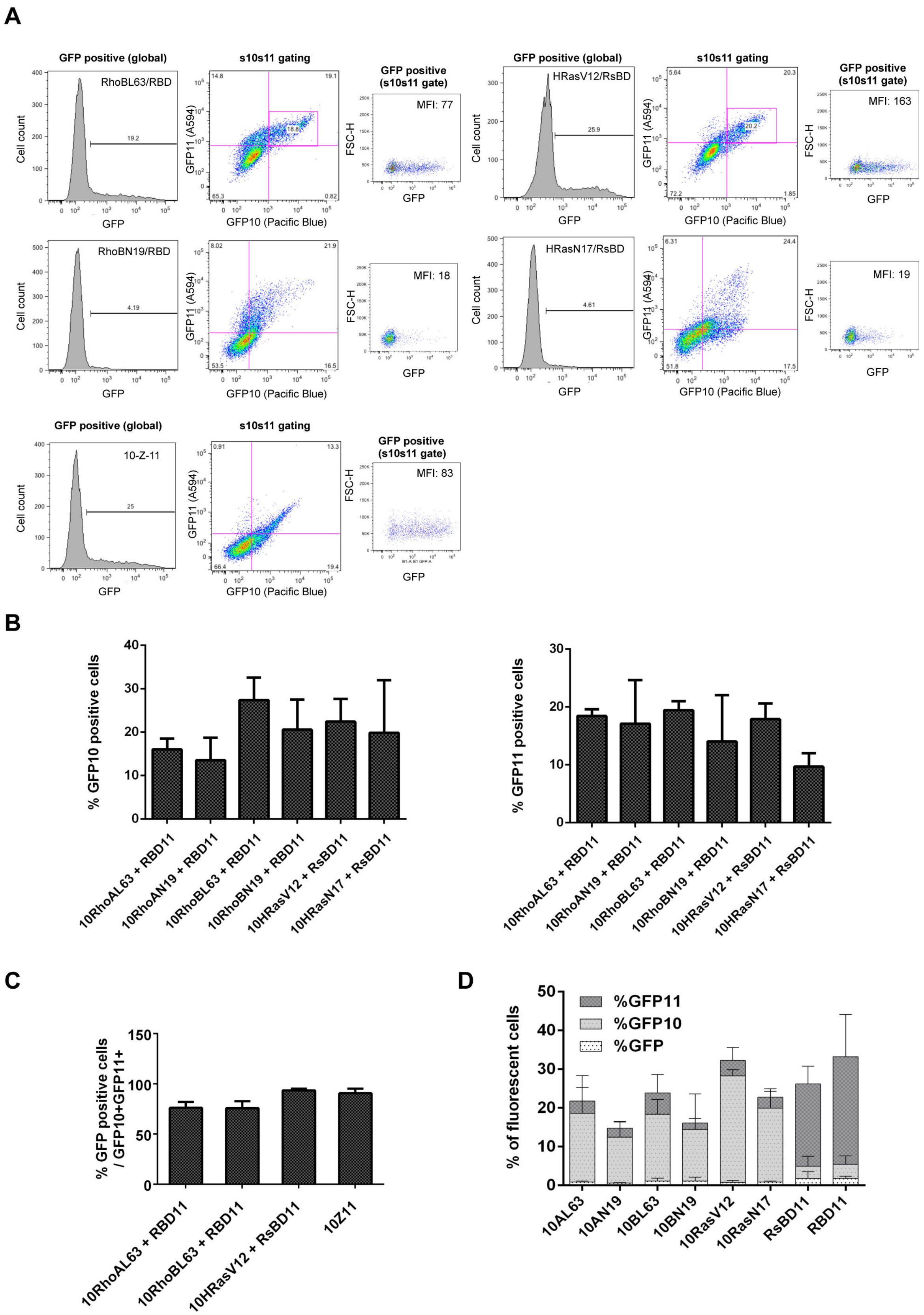

Journal of Cell Science • Supplementary information

Figure S2, related to Figure 1. FACS analysis controls for triSFP assays on Rho and Ras mutants. (A) FACS analysis histograms and dot plots for RhoB and HRas mutants. Representative dot plot of triSFP assays for RhoB/RBD and HRas/RsBD interactions for both constitutive and dominant negative GTPase variants, and for 10-Z-11 control. Both mean fluorescence intensities and GFP histograms in GFP10+GFP11+ co-expressing cells (s10/s11 gating) highlight the specificity of interaction towards GTP-state and a stronger binding of Ras to its effector domain than Rho. **(B)** Percentage of GFP10 and GFP11 positive cells for the indicated variants. Mean ± SEM; n=3 independent experiments. **(C)** Percentage of GFP positive cells in s10/s11 co-expressing cells for corresponding active Rho/RBD, active Ras/RsBD and for 10Z11 self-associating domain. Mean ± SEM; n=3 independent experiments. (**D)** Superimposed bar diagram showing quantification of split-GFP fluorescence (% GFP), GFP10 staining (% GFP10, Pacific Blue) and GFP11 staining (% GFP11, Alexa 594) of HEK1-9 cells transfected with GFP10 or GFP11 chimera and immunostained with both anti-GFP10 and anti-GFP11 antibodies. Mean ± SEM; n=3 independent experiments.

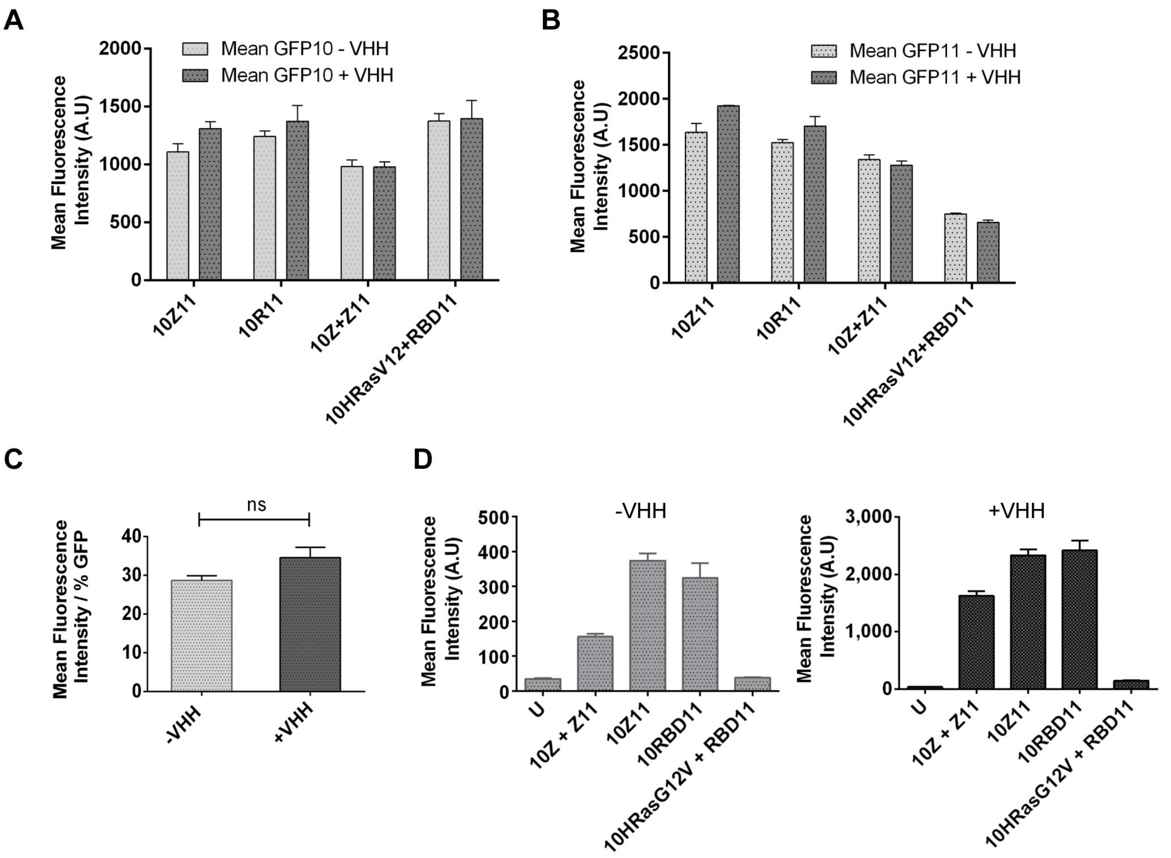

Figure S3, related to Figure 2. Control of split-GFP chimera expression on triSFP quantification with VHH booster. Expression of indicated split-GFP chimera constructs in MRC5_GFP1-9 cells (-VHH) and in MRC5_GFP1-9_VHH cells (+VHH). 10-Z-11 and 10-RBD-11 (10-R-11) GFP1-9 self-assocating domains, interacting leucine zippers (10-Z+Z-11), non interacting HRas/RBD (10HRasV12+RBD11)**.** **(A)** Mean fluorescence intensities of GFP10 positive cells (s10s11 gate). (**B)** Mean fluorescence intensities of GFP11 positive cells (s10s11 gate). **(C)** Mean fluorescence intensity of GFP transfected cells normalized by the percentage of GFP positive cells. t-test student, non significant. Mean ± SEM; n=3 independent experiments. **(D)** Mean Fluorescence intensity of rGFP in MRC5_GFP1-9 cells (-VHH) and in MRC5_GFP1-9_VHH cells (+VHH).

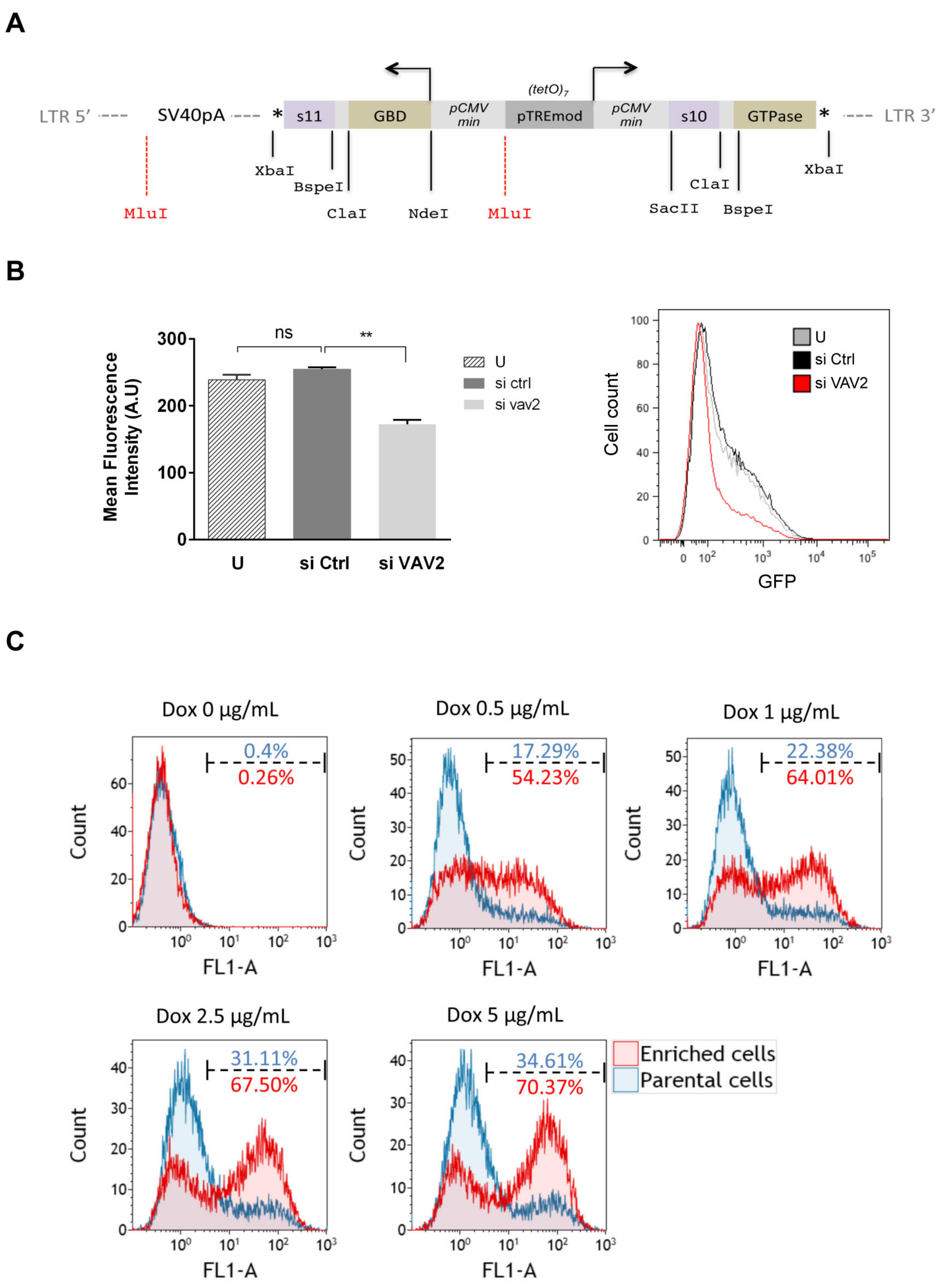

Figure S4, related to Figure 3 and 4. Design and characterization of the RhoB activation triSFP reporter cell model. (A) Cloning cassette of the pTRIP TRE BI promoter vectors. To inducibly express simultaneously GBD-11 and 10-GTPase chimera,

Journal of Cell Science • Supplementary information

the RBD-11 domain was cloned into the pTRE-Tight vector (Clontech). The DNA sequence encoding the minimal pCMV promoter and the SV40 polyA sequence was amplified by PCR and moved into the MluI site of a pTRIP TRE GFP10-RhoA or -RhoB vector (Vectoul CRCT, Toulouse France). Expression of both GFP10 and GFP11 chimera is achieved through the tet response element (TRE) composed of seven elements of the *tetO* promoter and pCMV minimal promoters (see. Gossen, M. & Bujard, H. (1992) Proc. Natl. Acad. Sci USA 89:5547–5551.). (**B)** Analysis of triSFP RhoB reporter fluorescence upon VAV2 GEF downregulation. TriSFP RhoB reporter cells were left untransfected (U), transfected with scrambled siRNA (si Ctrl) or with VAV2 siRNA (si VAV2) for 72h. RhoB/RBD expression was induced for 24h prior to GFP10 and GFP11 immunostaining. Mean GFP fluorescence intensity in GFP10+GFP11+ co-expressing cells for the three conditions tested. Mean ± SEM; n=3 independent experiments. Paired Student's t-test ns (non-significant), **P<0.05. (right image) Overlay of FACS histograms of GFP fluorescence in the corresponding GFP10/GFP11 positive cells. (**C)** Representative histograms overlay showing flow cytometry analysis of the triSFP RhoB activation reporter cell line (FL1-A, GFP channel) with increasing doxycycline concentrations for parental cells (blue) and cell-sorted cells (red). For each condition, the percentage of GFP positive cells upon doxycycline induction (upper right of the histogram) is defined to a control without doxycycline.

Journal of Cell Science • Supplementary information

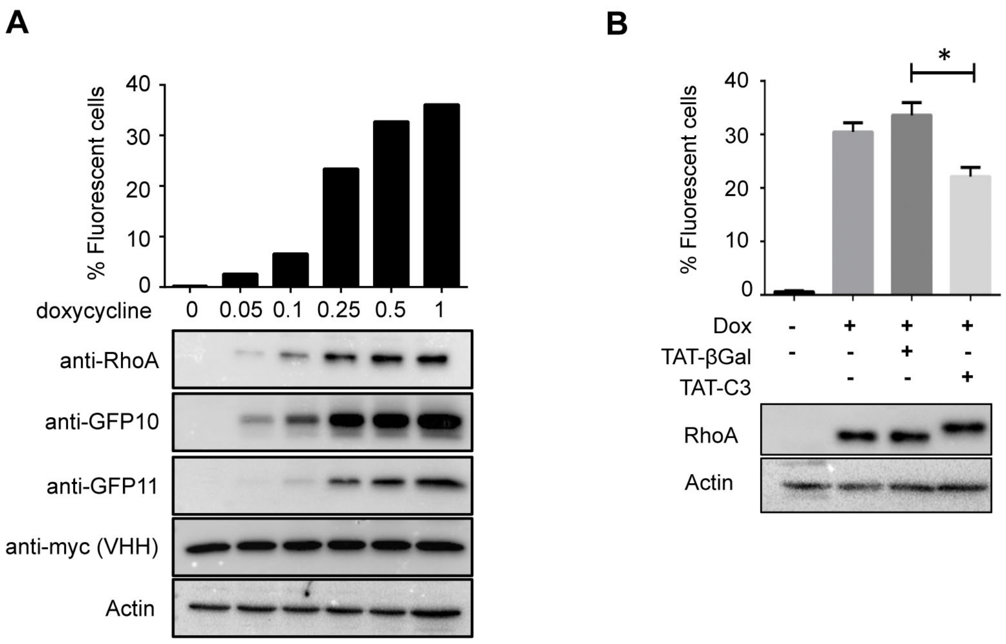

Figure S5, related to Fig. 5. Characterization of the RhoA activation triSFP cell model. **(A)** MRC5_GFP1-9 cells were transduced with pTRIP TRE BI 10-RhoA/RBD11 and VHH anti-GFP lentiviruses, sorted and characterized for expression of the various split-GFP chimera with increasing doxycycline dose-response: GFP10-RhoA was revealed with anti-RhoA and anti-GFP10 antibodies; RBD-GFP11 with anti-GFP11 and anti-GFP VHH with anti-myc antibodies. A concentration of 0.5 µg/mL doxycycline was chosen for the detection of RhoA activation modulation using FACS or fluorimeter analysis (**see Fig. 5**). **(B)** Inhibition of RhoA activation with TAT-C3 exoenzyme. FACS analysis of MRC5 triSFP RhoA activation reporter cells expressed for 24h with 10µg/ml TAT-β-Galactosidase control or 10µg/ml of TAT-C3 exoenzyme. Bar graph: mean ± SEM; n=3, Student's t-test $*P < 0.05$. The corresponding cells were lysed and submitted to Western blot analysis against RhoA and β-actin (one representative experiment).

Journal of Cell Science • Supplementary information

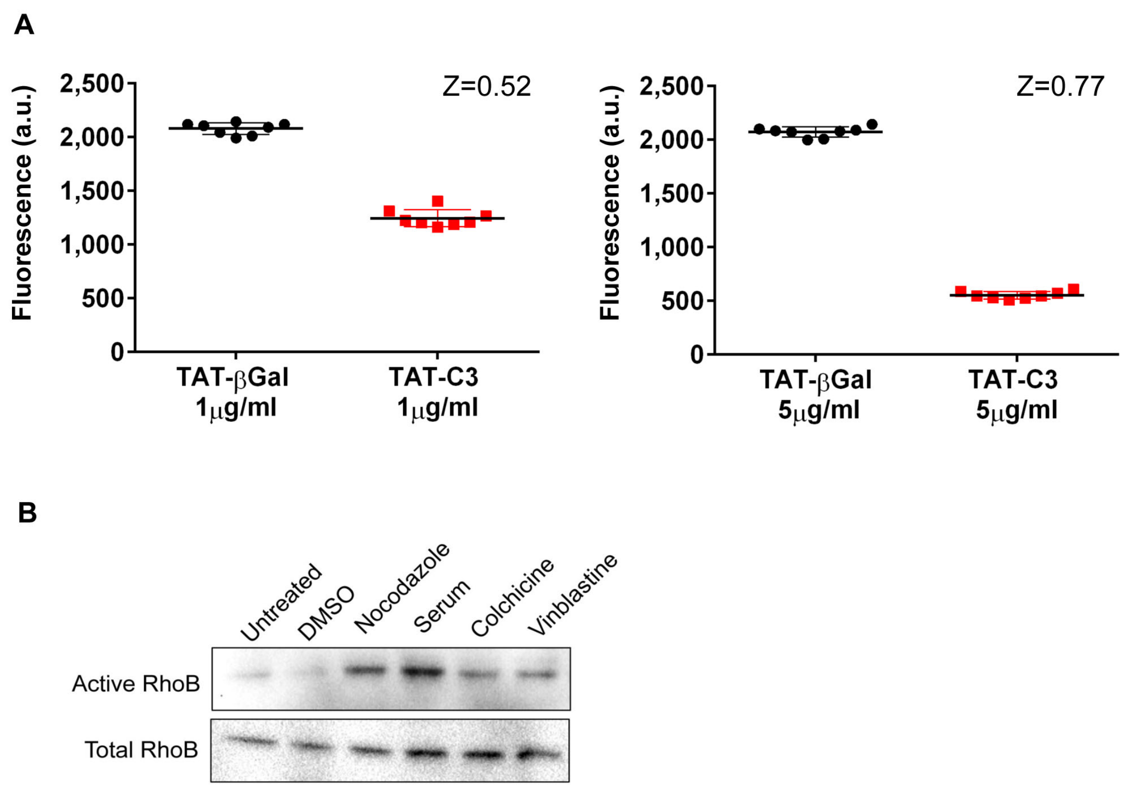

Figure S6, related to Figure. 6. High-content analysis of RhoB activation. (A) Calculation of Z factor for screening inhibitors of RhoB activation. RhoB triSFP reporter was treated simultaneously with doxycycline and TAT-C3 Rho inhibitor (1 and 5ug/ml) or with TAT-βGal control (1 and 5ug/ml). Fluorescence was measured on a Biotek fluorescence plate reader after 36 hours treatment. GFP fluorescence was substracted with background fluorescence value from uninduced control cells (mean of 6 replicates) and the Z-factor was calculated from the sample means and sample standard deviations of eight replicates as follow: 1- (3x ($SD_{TAT\text{-}C3}$+ $SD_{TAT\text{-}\beta Gal}$)/($Mean_{TAT\text{-}\beta Gal}$-$Mean_{TAT\text{-}C3}$). **(B)** Control of endogenous RhoB activation with microtubule destabilizing agents. Representative GST-RBD pulldown of active RhoB on HeLa cells treated with the indicated compounds for 30 min (5 μM Nocodazole, 10% serum, 1 μM Colchicine, 0.1 μM Vinblastine). Cells were serum-starved for 24 h prior treatment.

Journal of Cell Science • Supplementary information

Supplementary Table S1 . List of the plasmids used in the study.

Plasmids	Backbone
pTRE tight BI (Clontech)	Tet-on BI
pTRIP TRE BI GFP10RhoA/RBD11	Tet-on BI lentiviral
pTRIP TRE BI GFP10RhoB/RBD11	Tet-on BI lentiviral
pTRIP CMV GFP1-9	CMV lentiviral
pTRIP CMV GFP1-9 NES	CMV lentiviral
pTRIP CMV GFP1-9 NLS	CMV lentiviral
pTRIP CMV GFP1-9 CAAX	CMV lentiviral
pcDNA GFP10-RBD-GFP11	pcDNA 3.1
pcDNA GFP10-zipper-GFP11	pcDNA 3.1
pcDNA GFP10-zipper	pcDNA 3.1
pcDNA zipper-GFP11	pcDNA 3.1
pcDNA GFP10-RhoAQ63L	pcDNA 3.1
pcDNA GFP10-RhoAT19N	pcDNA 3.1
pcDNA GFP10-RhoBQ63L	pcDNA 3.1
pcDNA GFP10-RhoBT19N	pcDNA 3.1
pcDNA GFP10-HRasG12V	pcDNA 3.1
pcDNA GFP10-HRasS17N	pcDNA 3.1
pcDNA RBD-GFP11	pcDNA 3.1
pcDNA RsBD-GFP11	pcDNA 3.1

Supplementary Table S2 . List of the antibodies used in the study.

Primary antibodies	Supplier	Species	Type	Reference	Dilution	Use
**RhoA**	Santa Cruz Biotechnology	Mouse	Monoclonal	(26C4) sc-418	1/500	WB
**RhoB**	Santa Cruz Biotechnology	Rabbit	Polyclonal	(119) sc-180	1/3000	WB
**H-Ras**	Santa Cruz Biotechnology	Rabbit	Polyclonal	(C20) sc-520	1/1000	WB
**Actin**	Merck Millipore	Mouse	Monoclonal	Clone C4 MAB1501	1/20000	WB
**GFP11**	Agro-Bio	Mouse	Polyclonal	Agro-bio.com	1/1000	WB, FCS
**GFP11**	Agro-Bio	Rabbit	Polyclonal	Agro-bio.com	1/2000	WB
**GFP10**	Agro-Bio	Rabbit	Polyclonal	Agro-bio.com	1/2000	WB
					1/1000	IF
**GAPDH**	Cell signaling	Rabbit	Monoclonal	(14C10) 2118	1/3000	WB
**GFP (FL)**	Santa Cruz Biotechnology	Rabbit	Polyclonal	sc- 8334	1/200	IF
					1/1000	WB
**c-Myc**	Sigma-Aldrich	Mouse	Monoclonal	Clone 9E10 M4439	1/2000	IF
					1/5000	WB
**α-tubulin**	Sigma-Aldrich	Mouse	Monoclonal	Clone B-5-1-2 T5168	1/5000	WB, IF
**Vav2**	Cell signaling	Rabbit	Monoclonal	(C64H2) 2848	1/1000	WB

Secondary antibodies	Supplier	Reference	Dilution	Use
**Pacific Blue- conjugated anti Rabbit- IgG**	Life technologies	P10994	1/500	IF, FCS
**Alexa fluor 594 conjugate anti mouse IgG-**	Life technologies	A21125	1/500	IF, FCS
**Goat anti-mouse IgG (H+L)-HRP conjugate**	BIO-RAD	170-6516	1/3000-1/10000	WB
